# Editorial: Quality and nutrition of meat and meat products: emphasis on muscle protein structure, activity, modification, and functionality

**DOI:** 10.3389/fnut.2023.1301481

**Published:** 2023-11-13

**Authors:** Rui Liu, Tong Xing

**Affiliations:** ^1^School of Food Science and Engineering, Yangzhou University, Yangzhou, Jiangsu, China; ^2^College of Animal Science and Technology, Nanjing Agricultural University, Nanjing, Jiangsu, China

**Keywords:** muscle proteins, meat and meat products, protein interfacial properties, microbial quality, meat processing

Muscle proteins are highly nutritious and regarded as an important protein source in the human diet due to the abundance of essential amino acids. Muscle proteins play a decisive role in the overall properties of meat and meat products, including meat texture, tenderness, water-holding capacity, juiciness, and appearance. On the one hand, the structure, activity, and modification of proteins are responsible for biochemical events during the muscle-to-meat conversion process, which regulates the quality of raw meat. On the other hand, the functionality of myofibrillar proteins comprises capacities of gelling, emulsifying, and water binding and contributes to the quality and nutritional value of processed meat products. Various factors of postmortem storage and processing conditions, such as pH, ionic strength, oxidative and nitrosative stress, and heating, can affect the structure, activity, modification, and functionality of muscle proteins, thus influencing the final properties of meat and meat products. This Research Topic includes four articles (two review articles and two original articles). A summary of those studies is discussed as follows.

Emulsion-type products have attracted increasing attention for their various applications in nutraceuticals and functional foods. The quality of this type of product is largely determined by the emulsifier's interfacial behavior, among which proteins are one kind of major emulsifier in food. Zhang et al. gave a thorough review of oil-in-water (O/W) interfacial properties of proteins in two aspects: intrinsic factors and improving strategies. Muscle proteins (myoglobin, myosin, and myofibrillar proteins) and plant-based proteins (soy, pea, and whey protein) were mostly discussed. The emulsion-related protein characteristics included amphiphilicity, flexibility, primary sequence, aggregation, and protein concentration. The conformational flexibility of protein was considered the most determinate characteristic that influenced O/W interfacial properties because high flexibility could allow the protein to be more easily unfolded and adsorbed onto the interface or form a softer interfacial film. The physical and chemical approaches to improving protein interfacial properties were comprehensively summarized. The potential mechanisms included aggregate formation, reduced molecule size, amphiphilicity modification, increasing flexibility, co-adsorption and stabilization, and facilitating interfacial protein interactions. The need for further studies on interfacial–bulk protein interactions, the usage of *in silico* tools, the connection between interfacial–industrial processing properties, and the interfacial–sensory–digestive relationships of O/W emulsions was remarked on for developing emulsion-type products.

The qualities of meat and meat products are generally assessed by sensory evaluation, physical–chemical parameters, and microbial quantity. Bhawana et al. surveyed the qualities of raw chicken meat from various meat retail outlets in Hisar City, India. Variations were found in proximate composition, pH, color, water-holding capacity, and lipid oxidation of breast and thigh samples among six meat retail outlets. The control samples obtained from a college farm with standard procedures of the meat and meat processing laboratory had superior meat quality, especially microbial safety, exhibiting lower total plate and psychrotrophic counts than the chicken in the retail outlets. Though many factors, including personal unhygienic performance and environmental conditions during slaughter, storage, transportation, and selling, could affect the microbial quantity, it was highlighted that hygiene and meat safety were still regarded as severe challenges in developing countries. As such, a mini-review on the microbial diversity of meat and meat products under spoilage and its controlling approaches was conducted by Zhu et al. and included in this topic. The livestock and poultry meat spoilage phenotype included slime, gas production, biofilm formation, acid production, and discoloration. Bacterial genera, including *Pseudomonas spp., Bacillus spp., Crude Typhimurium spp., Schwartzella spp., and Aeromonas spp*., were considered as the dominant communities in cold meat and poultry packed under aerobic conditions. The practical strategies for controlling the microbial safety of meat and meat products were packing methods, the addition of antibacterial substances, plasma sterilization, bacteriophage sterilization, and low-dose irradiation. Moreover, due to the high nutritious value of meat that is favored by microbial growth and various contaminated sources during production, storage, transportation, and marketing, the diversity of microbial communities needs to be more comprehensively characterized to develop targeted approaches in industrial practice.

Vacuum-tumbling marination has been achieved as a common technology in meat processing. Ge et al. applied this technology in the processing of a local chicken breed, Xueshan chicken, and made a comparison with the traditional immersion method. It was found that the chicken that underwent vacuum tumbling had significantly higher absorptivity and meat tenderness than the chicken that underwent immersion treatment. In respect to the protein properties, vacuum tumbling decreased total sulfhydryl content alongside an increased free sulfhydryl content, protein surface hydrophobicity, and myofibril fragmentation index of myofibrillar proteins, indicating a greater extent of protein degradation and denaturation. Scanning electron microscopy revealed that vacuum tumbling destroyed the structure, increased the gap, and loosened the structure of the muscle fibers, with the authors thereby attributing this to the tenderness promotion.

## Author contributions

RL: Writing—original draft, Writing—review & editing. TX: Writing—review & editing.

